# Malignant Hypertensive Retinopathy in an Infant with Mid-Aortic Occlusion

**DOI:** 10.1155/2016/8162687

**Published:** 2016-10-05

**Authors:** Lawrence J. Oh, Gaurav Bhardwaj, David S. Winlaw, Craig E. Donaldson

**Affiliations:** ^1^Department of Ophthalmology, Save Sight Institute, Sydney, NSW, Australia; ^2^Sydney Medical School, University of Sydney, Sydney, NSW, Australia; ^3^Department of Ophthalmology, The Children's Hospital at Westmead, Sydney, NSW, Australia; ^4^The Heart Centre for Children, The Children's Hospital at Westmead, Sydney, NSW, Australia

## Abstract

*Purpose.* Case report describing an eight-month-old infant presenting with intermittent esotropia and irritability who was found to have malignant (grade 4) hypertensive retinopathy and mid-aortic syndrome.* Methods.* Visual acuity was 6/140 in the right eye and not recordable in the left eye. Blood pressure was as high as 230/120 mmHg. Fundoscopy revealed bilateral optic disc swelling, macular stars, and serous retinal detachment in the left eye, findings that are consistent with malignant (grade 4) hypertensive retinopathy. CT abdominal angiogram revealed a severe mid-aortic syndrome with occlusion of the abdominal aorta at T12.* Results.* The patient was treated with medical management of his hypertension, improving the subretinal exudate. Binocular visual acuity improved to 6/9.5 over 9 months. There was a persistent left relative afferent pupillary defect and moderate left esotropia.* Conclusion.* This is the first reported case of malignant hypertensive retinopathy in an infant with concomitant mid-aortic occlusion. The authors emphasize the need for an ophthalmological and pediatric examination in a child presenting with intermittent squint and irritability. The esotropia was found to be a false localizing sign of raised intracranial pressure secondary to the severe mid-aortic syndrome.

## 1. Introduction

Hypertensive retinopathy is uncommon in infants and is usually detected following ophthalmological referral in patients with known hypertension. Underlying renal and cardiac disease is a well-known cause of secondary hypertension in infants and children. Hypertension in infants and children has become increasingly prevalent with an estimate of 3.2–3.5% in the population [1]. The early signs of hypertensive retinopathy in children are behavioural changes, hypertension, and optic disc swelling [1–3]. If there is a delay in diagnosing hypertensive retinopathy, the risk of amblyopia may lead to a lifetime of visual impairment. We present a unique case of malignant hypertensive retinopathy due to life-threatening systemic vascular malformation, which initially presented to the ophthalmologist.

## 2. Case Presentation

An eight-month-old infant presented to an ophthalmologist with intermittent esotropia and irritability of two months duration. He had been a previously well baby, hitting the appropriate milestones for his age. The mother was healthy during pregnancy and delivered at full-term by Caesarian section.

On ophthalmic examination he had bilateral upper eyelid retraction. His visual acuity was approximately 6/140 in the right eye and not recordable in the left eye with Teller acuity. There was a left relative afferent pupillary defect and also a variable moderate angle left esotropia. His eye movements were full. There was rotatory nystagmus with a vertical duction. He was able to fix and follow objects with his right eye but not his left.

**Figure 1:**
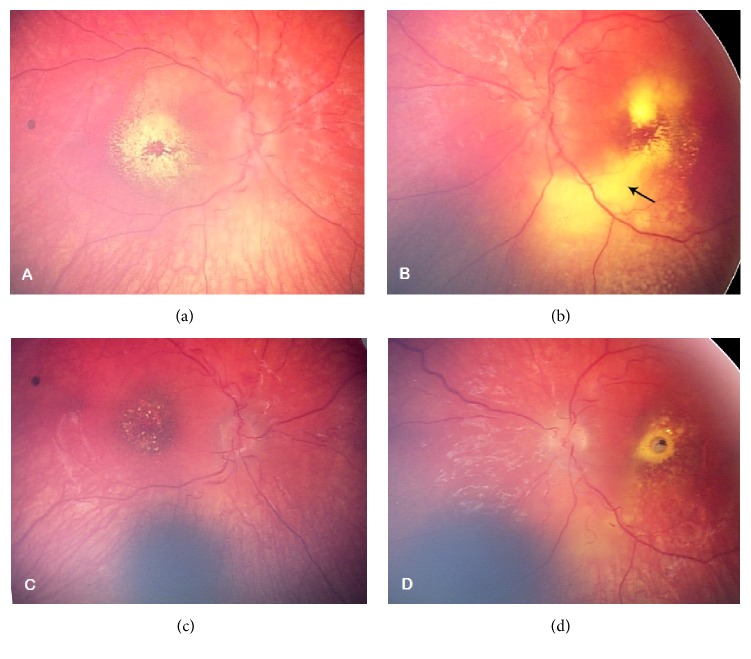
RetCam images. (a) Right eye. (b) Left eye. Bilateral optic disc swelling and macular star and left serous retinal detachment (arrow). (c) Right eye. (d) Left eye. Followup at 8 months shows reduced optic disc swelling and macular exudates and the suggestion of a macular pseudohole in the left eye.

On fundoscopy, there was evidence of bilateral optic disc swelling, vascular leakage (macular star), and secondary serous retinal detachment in the left eye (Figures 1(a) and 1(b)). This clinical appearance is consistent with malignant (Grade 4) hypertensive retinopathy.

He was admitted to a tertiary pediatric hospital for further investigation. In the emergency room, he was noted to be hypertensive with a blood pressure fluctuating between 140/100 and 230/120. Urgent neuroimaging (CT/MRI) showed gross communicating hydrocephalus, normal flow through the cerebral aqueducts, and no mass lesions. He was commenced on beta-blockers and calcium channel blockers for his hypertension.

On systemic examination, he was irritable but alert. A systolic heart murmur was present at the left lower sternal edge. His abdomen was soft and nontender, and his peripheral pulses, including brachial and femoral, were present and normal.

Blood investigations including complete blood count, electrolytes, and creatinine were unremarkable. Renal ultrasound was normal. Transthoracic echocardiography demonstrated concentric left ventricular hypertrophy, consistent with hypertension, and there was no evidence of aortic coarctation.

**Figure 2:**
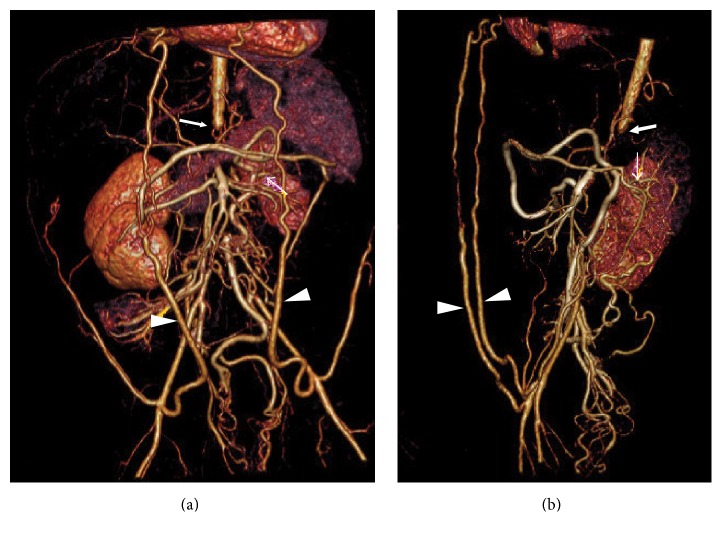
CT angiogram (3D reconstruction): (a) anterior; (b) lateral. The abdominal aorta is occluded at T12 (thick arrow). Both internal mammary arteries are markedly enlarged and ramify with the inferior epigastric arteries (arrowheads). The renal arteries arise from collaterals inferior to the termination of the aorta. The left renal artery (thin arrow) is small and there is decreased enhancement of the anterior half of the left kidney.

An abdominal angiogram (Figure 2) demonstrated a severe mid-aortic syndrome, with occlusion of the abdominal aorta at T12. The left renal artery was small and there was decreased enhancement of the anterior half of the left kidney.

The patient's symptoms improved with medical management of his hypertension. Followup examination of his eyes showed improvement of his subretinal exudate (Figures 1(c) and 1(d)). His binocular visual acuity improved from 6/80 to 6/9.5 over the next 9 months. His left visual acuity continued to be unrecordable, raising the possibility of a left ischaemic optic neuropathy. He was able to fix and follow with his right eye but had a persistent left relative afferent pupillary defect. He continued to demonstrate moderate left esotropia, more marked on accommodation.

## 3. Discussion

To our knowledge, this appears to be the first reported case of malignant hypertensive retinopathy in an infant and the first in association with a complete mid-aortic occlusion. In a previous case series of children with hypertension, the rate of retinopathy was 6–51% [1–3] and the severity was reported as mild in the majority of cases with the youngest patient being two years old.

Hypertension in infants is usually related to renal disease including renal artery thrombosis or stenosis or vascular disease including coarctation of the aorta. The evaluation and management should be prompt with moderate reduction of blood pressure to a level below the 95th percentile in order to avoid end-organ damage [4]. This is best commenced in a pediatric tertiary hospital setting with input from multiple medical teams.

Generally, management involves antihypertensive therapy to gradually reduce blood pressure due to the reactive retinal arteriolar narrowing, thus maintaining end-organ perfusion including the optic nerve. Conversely rapid reduction in blood pressure can cause visual impairment attributed to optic nerve infarction [4, 5].

This infant has a severe form of midaortic syndrome and will eventually require an operation to provide an alternative pathway for blood to travel from the proximal thoracic aorta to the iliac vessels, with connection of the renal and splanchnic vessels. This procedure is delayed as long as possible to allow somatic growth, while the systemic blood pressure is medically controlled [6].

In infants with swollen optic discs, urgent neurological examination and neuroimaging are required to exclude papilloedema. Although no cerebrospinal fluid pressure was measured in this patient, the esotropia may have been a false localizing sign of raised intracranial pressure. There may also have been a sensory component to the esotropia [7]. Other differential diagnoses for retinal exudation and lipid include neuroretinitis and Coats disease, which are usually unilateral or asymmetric. Exudative retinal tumors also need to be borne in mind such as retinoblastoma or retinal capillary hemangioma although their appearance is different than this patient.

This case reminds us of the importance of a thorough ophthalmic exam in children presenting with intermittent squint and irritability. Retinal photography may be beneficial in detecting subtle signs, documenting findings, and monitoring progress. In this case, the fundus findings would also have been identified by fundoscopy [8]. The ophthalmologist was the first medical specialist to see the patient presenting with a squint and nystagmus. However, the need for pediatric review and measurement of blood pressure in such cases should be emphasized to consider underlying systemic conditions.
